# Intergenus F1-hybrids of African weakly electric fish (Mormyridae: *Gnathonemus petersii ♂* × *Campylomormyrus compressirostris* ♀) are fertile

**DOI:** 10.1007/s00359-022-01542-5

**Published:** 2022-02-04

**Authors:** Yevheniia Korniienko, Kingsley C. Nzimora, Marianne Vater, Ralph Tiedemann, Frank Kirschbaum

**Affiliations:** 1 Faculty of Life Sciences, Albrecht Daniel Thaer-Institute of Agricultural and Horticultural Sciences, Unit of Biology and Ecology of Fishes, Humboldt University of Berlin, Philippstr. 13, Haus 16, 10115 Berlin, Germany; 2 Institute of Biochemistry and Biology, Unit of Evolutionary Biology/Systematic Zoology, University of Potsdam, Karl-Liebknecht-Str. 24-25, Haus 26, 14476 Potsdam, Germany; 3 Institute of Biochemistry and Biology, Unit of General Zoology, University of Potsdam, Karl-Liebknecht-Str. 24-25, Haus 26, 14476 Potsdam, Germany

**Keywords:** Mormyridae, *Campylomormyrus*, F1-hybrids, Ontogeny, Electric organ discharge

## Abstract

Hybridisation is an important element of adaptive radiation in fish but data are limited in weakly electric mormyrid fish in this respect. Recently, it has been shown that intragenus hybrids (*Campylomormyrus*) are fertile and are able to produce F2-fish. In this paper, we demonstrate that even intergenus hybrids (*Gnathonemus petersii* ♂ × *Campylomormyrus compressirostris* ♀) are fertile. Three artificial reproduction (AR) trials, with an average fertilisation rate of ca. 23%, yielded different numbers of survivals (maximally about 50%) of the F1-hybrids. The complete ontogenetic development of these hybrids is described concerning their morphology and electric organ discharge (EOD). Two EOD types emerged at the juvenile stage, which did not change up to adulthood. Type I consisted of four phases and Type II was triphasic. The minimum body length at sexual maturity was between 10 and 11 cm. Malformations, growth and mortality rates are also described.

## Introduction

The African weakly electric mormyrids comprise more than 200 species in 21 genera (Alves-Gomes and Hopkins 1997; Ozouf-Costaz et al. 2015; Sullivan and Lavoué 2015; Nelson et al. 2016). Their electric organ discharge (EOD) is species specific and shows a high diversity across the species (see e.g., Hopkins 1981; Hopkins and Bass 1981; Lamanna et al. 2016). This diversity is in part based on structural characteristics of the electric organs (EO) (Bennett and Grundfest 1961; Bass 1986). Nagel et al. (2017) showed that differential expression of voltage gated ion channel genes also has an impact on the EOD duration.

The mormyrid genus *Campylomormyrus* encompasses 15 species (Taverne 1972; Feulner et al. 2007), most of which are endemic to the Congo River basin (Feulner et al. 2007, 2008, 2009b; Lamanna et al. 2016; Kirschbaum et al. 2016). Species of this genus are characterised by very diverse EODs that differ in shape and duration from ca. 200 µs (as in *C. compressirostris*) to ca. 25 ms as in *C. rhynchophorus* and *C. numenius* (Feulner et al. 2008, 2009a, 2009b; Nguyen et al. 2020). In addition, there are basic differences in the anatomy of the electrocytes of the EO concerning the organisation and location of the stalk system, which consists of numerous small specialised protrusions of the electrocyte that fuse to form the main stalk, which receives the innervation by electromotor neurons. In *C*. *tamandua*, the main stalk is located at the rostral face of the electrocyte and the small stalks penetrate the electrocyte from the caudal to the rostral face (Paul et al. 2015). This feature correlates with the presence of an initial head negative phase in the EOD (Bass 1986; Gallant et al. 2011). In contrast, in the four species *C. compressirostris*, *C. tshokwe*, *C. numenius* and *C. rhynchophorus* there is no initial head negative phase in the EOD; the main stalk is found on the caudal face of the electrocyte and penetrations are absent (Paul et al. 2015; Nguyen et al. 2020).

Hybridisation is an important element in adaptive radiation and speciation in many taxa including fish (e.g., Selz and Seehausen 2019) but data on naturally occurring hybrids in weakly electric fish are scarce (Sullivan et al. 2004; Arnegard et al. 2005; Gallant et al. 2011). Using artificial reproduction (AR), Kirschbaum et al. (2016) showed that intragenus hybrids of *Campylomormyrus*, which combine genotypes coding for different EOD types, are fertile and able to produce vital offspring. They investigated eight intragenus hybrid types, produced between five species of the genus *Campylomormyrus* (*C. compressirostris*, *C. tshokwe*, *C. rhynchophorus*, *C. numenius* and *C. tamandua*), concerning the ontogeny of anatomical features of the EOs and the EODs, including duration and waveform. When a species with a short EOD was crossed with a species with a long duration EOD, the longer EOD always dominated in the EOD of the hybrid. Effects on EOD waveform were divers. The hybrid between *C. rhynchophorus* and *C. numenius* showed that the biphasic EOD of *C. numenius* dominated over the triphasic EOD of *C. rhynchophorus*. Of particular interest were the hybrids between *C. tshokwe* and *C. compressirostris* and *C. tshokwe* and *C. tamandua*, respectively. The EODs of *C. compressirostris* and *C. tamandua* are short and do not change during ontogeny. In contrast, the EOD of *C. tshokwe* undergoes a considerable change over several weeks during ontogeny, with a conspicuous double head positive waveform in juveniles at a TL of 46 mm (Nguyen et al. 2017). This specialised EOD waveform is also produced by the two hybrids, but at a later stage of ontogeny (Nzimora 2020).

Interestingly, all hybrids between *C. tamandua* (rostral position of the main stalk and penetrations) and those species with the caudal position of the main stalk and missing penetrations (*C. compressirostris*, *C. tshokwe*, *C. rhynchophorus* and *C. numenius*) exhibited an EOD with an initial head positive phase. In these hybrids, the main stalk was located at the caudal face, however, as a new feature, double penetrations occurred, which were not observed in any of the parents.

In addition to these intragenus *Campylomormyrus* hybrids, intergenus hybrids were produced (Kirschbaum et al. 2016), namely between *Campylomormyrus* species and *Gnathonemus petersii*. *Gnathonemus* is the sister genus of *Campylomormyrus* (Sullivan et al. 2000; Lavoué et al. 2003; Sullivan and Lavoué 2015) and occurs sympatrically with the *Campylomormyrus* species in the lower Congo River system (Gosse 1984). The EO of *Gnathonemus petersii* resembles that of *C. tamandua* in that the main stalk is located rostrally and penetrations occur (Bruns 1971); the discharge represents a tetraphasic EOD (Kramer and Westby 1985; Terleph and Moller 2003).

The structure of the EO of the intergenus hybrid between *G. petersii* and *C. rhynchophorus* differed from that of the intragenus hybrid between *C. rhynchophorus* and *C. tamandua*. Similar to the intragenus hybrids, the stalk was located caudally but penetrations did not occur (Kirschbaum et al. 2016). Thus, in this hybrid only the structural features of the EO of the *Campylomormyrus* species were expressed, whereas those of *G. petersii* did not appear. To find out, if this characteristic is related to the genetic inventory of *G. petersii*, an additional hybrid between another *Campylomormyrus* species (*C. compressirostris*) and *G. petersii* was produced (Kirschbaum et al. 2016), but so far only the first steps of the ontogeny of this hybrid, concerning the EO, were described. The aim of the current study, therefore, is a detailed description of the ontogeny of the EO and, in addition, to unravel whether these intergenus hybrids are fertile, as has been observed in intragenus *Campylomormyrus* (*C. tamandua* × *C. compressirostris*) hybrids (Kirschbaum et al. 2016; Korniienko et al. 2020).

## Materials and methods

### Breeding experiments including artificial reproduction

Hybrids between male *G. petersii* and female *C. compressirostris* were generated by AR, a technique already described for several mormyrid species (Nguyen et al. 2017) and for intragenus (*Campylomormyrus*) (Kirschbaum et al. 2016; Korniienko et al. 2020) and intergenus hybridisations (Kirschbaum et al. 2016). The two parental species were imported from Africa to Germany via a wholesale dealer. Preceding the AR, the development and maturation of the gonads had to be stimulated by imitation of rainy season conditions, i.e., by lowering water conductivity over several weeks (Kirschbaum 1987, 2006; Kirschbaum and Schugardt 2002b, 2006; Nguyen et al. 2017).

Temperature in the breeding tanks ranged from 23 to 27 °C, pH from 5.2 to 7. Temperature and water conductivity were measured daily with a conductivity meter (WTW Multi 340i; WTW Wissenschaftlich–Technische Werkstätten, 82362 Weilheim, Germany). pH was measured once a week with a pH meter (WTW Multi 340i; with the PH electrode SenTix 41; WTW Wissenschaftlich–Technische Werkstätten, 82362 Weilheim, Germany). Photoperiod was held constant at 12L:12D (light from 6:00 a.m. to 6:00 p.m.) and was controlled by a time switch.

During the AR, four males of *G. petersii* and three females of *C. compressirostris* were used (Table 1). A gonadotropin releasing hormone (GnRH) (commercial name Ovaprim: GnRH + Domperidone; Western Chemical Inc., Ferndale, WA 98248, USA) was injected into the dorsal lateral muscle of the fish (after it had been anaesthetised by Ethylenglycolmonophenylether, 0.2 ml per 1 l of water) to stimulate ovulation and sperm release. The GnRH was diluted 1:5 in physiological saline solution and used at a dosis of 25 μl/10 g of body weight.

**Table 1:** Comparative data of the three artificial reproduction (AR) trials between *Gnathonemus petersii* ♂ and *Campylomormyrus compressirostris* ♀ concerning fertilisation rate, mortality during embryonic development, hatching rate, proportion of malformations during the free embryonic stage and survival rate

No. of artific. reprod. (AR)	No. of ♂ used in AR (TL, cm)	No. of ♀ used in AR (TL, cm)	No. of eggs	No. of fertilised eggs (%)	No. of dead embryos during embryonic stage (%)	No. of hatched embryos (%)	No. of free embryos with malformations (%)	No. of surviving juvenile resp. adult fish (%)
1	1 (ca. 21)	1 (16)	161	36 (22.4)	5 (13.9)	31 (86.1)	0 (0)	1 (2.8)
2	2 (19/26.5)	1 (16)	170	43 (25.3)	1 (2.3)	42 (97.7)	1 (2.4)	0 (0)
3	1 (20)	1 (16)	309	62 (20.1)	5 (8.1)	57 (91.9)	9 (15.8)	30 (48.4)
Average	–	–	213	47 (22.6)	4 (8.1)	43 (91.9)	3 (6.1)	10 (17.1)

The number of fish used in the trials and their total length (TL) are included

About 24 h after the hormone injection, the specimens were anaesthetised again, as described above. The abdominal region of the females was dried and the eggs were carefully pressed out by hand and released into clean dry Petri dishes. The same procedure was repeated with the males: the sperm liquid was put into the Petri dishes next to the eggs. Subsequently, a small amount of water from the breeding tank was added into the Petri dish and the gametes were gently mixed. The Petri dishes with the inseminated eggs were put into an incubator with a temperature of 27 ± 0.5 °C and all eggs were checked visually for fertilisation in the next 3–5 h (binocular microscope Leica S6E). The unfertilised eggs were removed from the Petri dishes.

### Rearing conditions

Three hours after AR all eggs were checked: those with blastodern development (fertilised eggs) were separated from those with just protoplasm on top of the yolk (unfertilised eggs). Both, fertilised and unfertilised eggs were checked again ca. 16 h after AR and also later on with a binocular microscope and controlled for abnormalities and/or malformations. If development of the fertilised eggs stopped during the 3 days of embryological development they were classified as dead embryos (see Table 1) and they were removed from the Petri dishes with the live embryos. Five to ten fertilised eggs were kept per Petri dish (ø = 8.6 cm, *h* = 1.5 cm) for the 3 days of embryonic development. If necessary, the water in the Petri dishes was replaced by water from the breeding tank.

After hatching, on day 3, the free embryos were put into new Petri dishes and controlled for malformations; malformed free embryos were kept individually. Larvae, after the beginning of exogenous feeding on day 11, were kept in quadrangular Petri dishes (10 × 10 × 2 cm) up to day 30 of their development. The larvae were fed on freshly hatched *Artemia* nauplii twice a day and small Cladocera were supplied as well.

During the further larval and early juvenile stages, the fish were kept in plastic boxes of 20 × 10 × 5 cm equipped with aeration and hiding-places, such as small parts of broken plant pots and PVC pipes of a diameter of about 3 cm. Up to this point, the larvae were raised at room temperature of about 23–25 °C. Five fish were raised individually to follow their ontogenetic development. Older specimens were kept in plastic boxes at a size of 22 × 11 × 5 cm in a density of five to seven fish per box. At a total body length (TL) of about 40 mm, the fish were transferred into small tanks of 30 × 20 × 20 cm equipped with aeration, hiding-places and gravel of 2–3 cm in diameter. Snails and small shrimps devoured the uneaten food. At an age of about 170 days, the specimens were transferred into larger tanks (100 × 40 × 40 cm) with a density of ten specimens per tank. The individually kept specimens with TLs of about 68–80 mm were placed into bigger tanks (75 × 70 × 60 cm). Juveniles beyond 40 mm of a TL were fed on small (2–4 mm) live chironomid and *Chaoborus* larvae. Adult fish were fed twice daily with live white *Chaoborus* (*Corethra*) larvae and live chironomids. Frozen food was offered when live larvae were not available.

### Measurements and collection of data

Photos of eggs, free embryos and up to 15 mm long larvae were taken with a Leica S6E stereo zoom microscope fitted with a Canon Powershot S50 digital camera. Older larvae, juveniles and adults were photographed with digital cameras Canon EOS 350D or Canon EOS 100D.

The EODs were recorded using a Tektronix TDS 3012B digital phosphor oscilloscope (maximum sampling rate 1.25 GS/s; 9 bit vertical resolution) connected to two different differential preamplifiers. Larvae up to 20 mm of a TL were recorded with a Tektronix ADA 400A differential preamplifier (variable gain from 0.1 × up to 100x; bandwidth 100 Hz–1 MHz) and larvae beyond 20 mm of a TL, juveniles and adults with a Tektronix TM 502A differential amplifier (gain 10 V; upper bandwidth 3 kHz). The oscilloscope and preamplifiers were AC coupled.

Larvae with a TL up to 30 mm were retained in a small cube-like plastic box of 7.5 × 5 × 4 cm with a water level of 1.5 cm; bigger larvae, juvenile and adult fish were placed in a bigger plastic container of 30 × 15 × 15 cm with water levels from 3 to 10 cm. Water conductivity (during measurements) ranged from 600 to 800 µS/cm, temperature from 23 to 25 °C and pH from 6 to 8. The plastic containers were equipped with an adjustable compartment to prevent movements of the fish. Steel electrodes were positioned a few centimetres from the head of the fish (positive electrode) and near the tail (negative electrode). To reduce the stress during recording, the measurements were usually performed in darkness or at low light conditions. The EODs were recorded twice per month for the larvae, once per month for juveniles up to 70 mm, once per 3 months for adult specimens.

## Results

### Hybridisation experiments between male *G. petersii* and female *C. compressirostris*

We performed three AR experiments (experiment I from date 10.08.2016, experiment II–from 22.11.2016, experiment III–05.12.2017) with male *G. petersii* and female *C. compressirostris.* From three individual females of 16 cm of a TL, used in these experiments, we obtained 161, 170 and 309 eggs, respectively (Table 1). The number of fertilised eggs ranged between 20.1 and 25.3%. The majority of embryos (86.1–97.7%) hatched and only few showed malformations (Table 1).

### Ontogenetic development

**Fig. 1:**
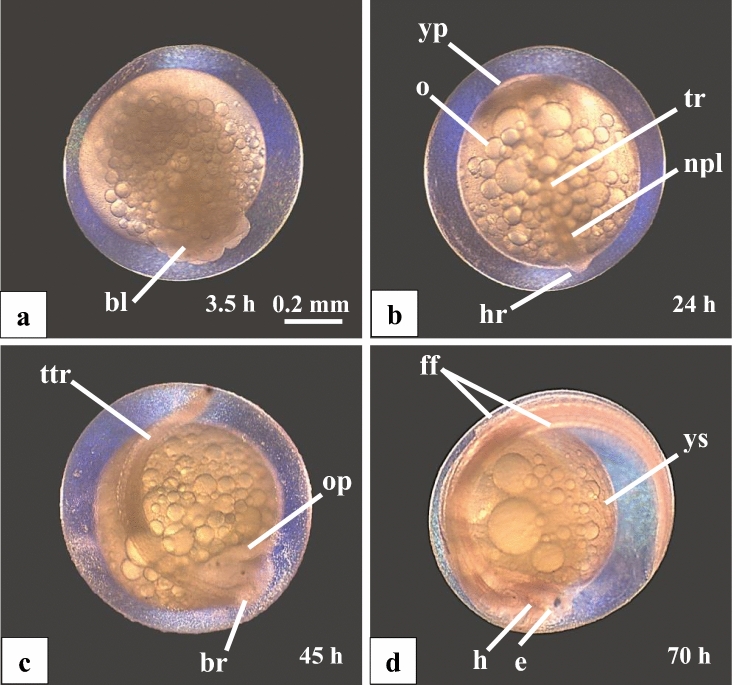
Embryogenesis of F1-hybrids between *Gnathonemus petersii* ♂ and *Campylomormyrus compressirostris* ♀. **a** 3.5 h old egg with a few blastomeres (bl) on top of the yolk. **b** 24 h after fertilisation, the outlines of the future embryo are apparent: the head region (hr) with the neural plate (npl), the trunk region (tr) and the yolk plug (yp). Oil droplets (o) are evenly distributed in the yolk. **c** 45 h old embryo possesses a well-developed head with large brain structures (br) and an otic placode (op). The trunk-tail region (ttr) shows segmented myotomes. **d** 70-h-old embryo shows a large head (h) with pigmented eyes (e). On the tail, the embryological fin fold (ff) is visible. The size of the yolk sack (ys) has decreased. The scale bar applies to **a**–**d**

A few hours after insemination, the first blastomeres are visible on top of the yolk (Fig. 1a). In the 1-day-old egg, the head and trunk regions of the embryo are detectable (Fig. 1b). A day later, the embryo’s brain is clearly outlined as well as the otic placode, the tail and individual myotomes (Fig. 1c). First movements of the tail were observed. On day 3, the embryo is ready to hatch. It features an embryonic fin fold in the caudal trunk and tail region, the tail is elongated and in the head region, small eyes are formed (Fig. 1d). The first free embryos started to hatch 70–72 h after fertilisation.

**Fig. 2:**
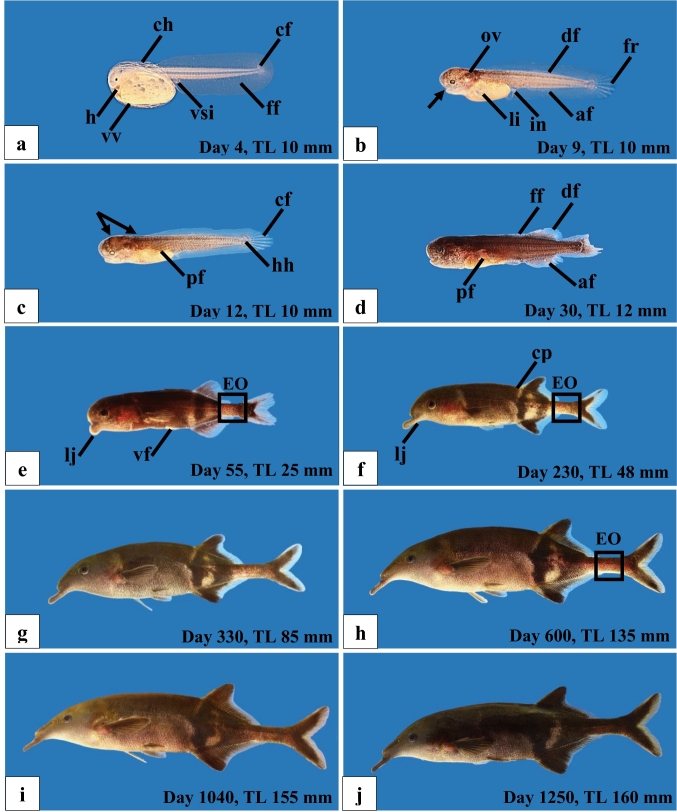
Ontogenetic development of F1-hybrids between *Gnathonemus petersii* ♂ and *Campylomormyrus compressirostris* ♀ from the free embryo on up to the adult fish. **a** Free embryo on day 4, just after hatching. Remnants of the chorion (ch) are visible. The heart (h), the venae vitellinae (vv) and the vena subintestinalis (vsi) are visible. The large embryological fin fold (ff) contains the caudal fin mesenchyme (cf). **b** Nine-day-old free embryo. The mouth has opened (black arrow). The otic vesicle (ov) is visible behind the pigmented eye. The liver (li) can be identified inside the yolk sack and the differentiation of the intestine (in) is externally visible. In the embryological fin fold, the mesenchyme of anal (af) and dorsal fin (df) are just apparent; the fin rays (fr) of the caudal fin are visible. **c** 12-day-old larva at the beginning of exogenous feeding. Melanophores (double arrow) originate in the head region and distribute over the body. A large pectoral fin (pf) is apparent and the hypural plates (hh) of the caudal fin skeleton are seen. The caudal fin (cf) is supported by thick fin rays. **d** At day 30 the larva is now evenly brown coloured, the median fin fold (ff) is largely reduced, the unpaired fins (df, dorsal fin; af, anal fin) and pectoral fin (pf) are well developed. **e** An early juvenile at day 55 with well-developed paired ventral fins (vf). The positive allometric growth of the lower jaw (lj) starts. The tail region is characterised by the formation of a distinct peduncle, which contains the adult EO (black rectangle) as shown in the juvenile (**f**) and adult fish (**h**). A striped pigment pattern (cp) develops at the rear part of the body and is clearly identifiable in (**f**) showing a 230-day-old juvenile. Note the continuous prolongation of the lower jaw (lj) in the 48 mm long juvenile (**f**). Late juvenile (**g**) and adult fish (**h–j**) show progressive increase in body height and length

Figure 2a shows a free embryo after hatching on day 4. By this time, more details are visible: the heart below the head and blood vessels surrounding the yolk. The first fin rays of the caudal fin start to differentiate (Fig. 2a). On day 9 (Fig. 2b), the eyes have increased in size, the mouth has opened, the prospective anus is apparent, in the fin fold mesenchymatic tissue of the dorsal and ventral fins is detectable and the fin rays of the caudal fin are well developed. Pigmentation starts in the head region. The larva at the beginning of exogenous feeding (start on day 11) is shown in Fig. 2c. The yolk sac is almost completely reduced, indicating the end of the free embryonic stage and the start of the larval stage. Pigmentation extends in the trunk region (arrows), the pectoral fins have developed and supporting bony plates of the caudal fin are detectable. On day 30 (Fig. 2d), dorsal and anal fins are well formed, but remnants of the embryological fin fold are still present, indicative of the larval stage. The body is evenly pigmented with numerous small melanophores. Note that the displayed larva has lost a part of the caudal fin and shows injuries on the dorsal and pectoral fins, due to aggressive attacks of other larvae in the community tank.

The juvenile stage started at a TL of about 21 mm in 60–65 days old fish, when the fish are characterised by a full regression of the embryological fin fold and produces the typical juvenile EOD. Figure 2e shows an early juvenile with well-developed unpaired and paired fins, which are still mostly transparent. The body features distinct dark and light pigment patterns. Jaw anatomy starts to change with a slight prolongation of the lower jaws. The tail region is characterised by the formation of a distinct peduncle, which contains the adult EO. Figure 2f–g shows later stages of the juvenile development. Body height increases and the positive allometric growth of upper and lower jaws continues. In the unpaired fins more and more melanophores develop. In the adult fish (Fig. 2h–j), growth continues, but without a change in body proportions. A comparison of adult F1-hybrids with the parent species is provided in Fig. 8. In the F1-hybrids, the morphology of the snout is intermediate between the two parental species, whereas the pigment pattern more closely resembles that of *G. petersii*. The paternal species *G. petersii* possesses brightly coloured stripes on the caudal part of the body, near the dorsal and anal fins, which first appear at a TL of about 18 mm (Korniienko unpublished data). A similar colouration was found in another intergenus hybrid between *G. petersii* and *C. rhynchophorus* (Elarbani 2017).

### Malformations, abnormalities and mortality

**Fig. 3:**
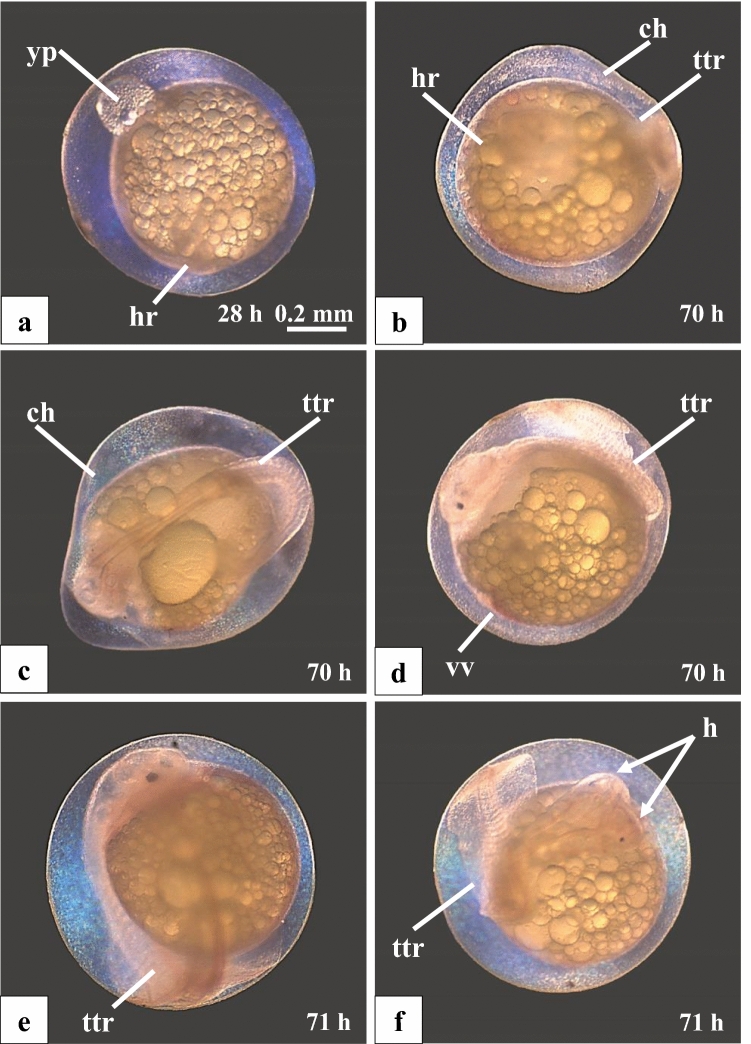
Examples of malformed F1-hybrid embryos (*Gnathonemus petersii* ♂ × *Campylomormyrus compressirostris* ♀). **a** 28-h-old embryo with abnormally developed yolk plug (yp) and underdeveloped head region (hr). **b**–**d** 70-h-old embryos with various kinds of deficiencies like undeveloped head region (hr), deformed trunk-tail region (ttr), abnormal venae vitellinae (vv) and a deformed chorion (ch). **e, f** 71-h-old embryos with trunk-tail deformations (ttr) and with two heads (h, *double arrows*) (**f**). Scale bar applies to (**a–f**)

Malformations were already observed in some of the hybrid offspring about 28 h after fertilisation; e.g., an abnormally developed yolk plug on top of the yolk (Fig. 3a). Other types of malformations were observed ca. 70 h after fertilisation. Some embryos had underdeveloped head regions (Fig. 3a, b). Others showed various degrees of deformations of the trunk-tail region (about 70–80% of all malformations) (Fig. 3c–f), often in combination with other kinds of defects, such as an enormously developed yolk sac or deficiencies of the circulatory system. One embryo developed two heads (Fig. 3f). About 30% of the malformed embryos died in the pre-hatching stage.

**Fig. 4:**
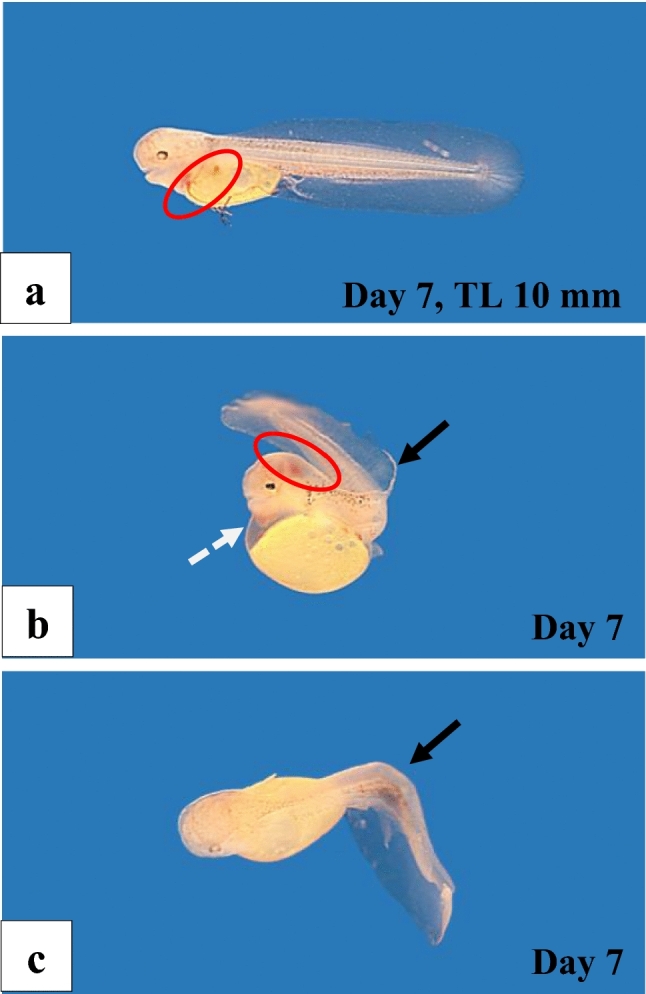
Examples of malformed F1-hybrid free embryos (*Gnathonemus petersii* ♂ × *Campylomormyrus compressirostris* ♀), 8 days old. **a** Embryo with abnormally developed circulatory system (*red circle*). **b** Embryo with a malformed yolk sack (*white arrow*), a distorted vertebral column (*black arrow*) and an abnormal blood supply in the head region (*red circle*). **c** Embryo with heavily deformed vertebral column (*black arrow*)

Animals with malformations that had survived to the free-embryo stage died between day 7 and day 10 after fertilisation. The malformations included an abnormally developed circulatory system in rostral parts of the body (Fig. 4a) and deformations of the yolk sac and the vertebral column (Fig. 4b, c). The percentage of malformations during the embryonic and free embryonic stage varied among the three experiments and reached a maximum of 17.4% in experiment III (Table 1).

**Fig. 5:**
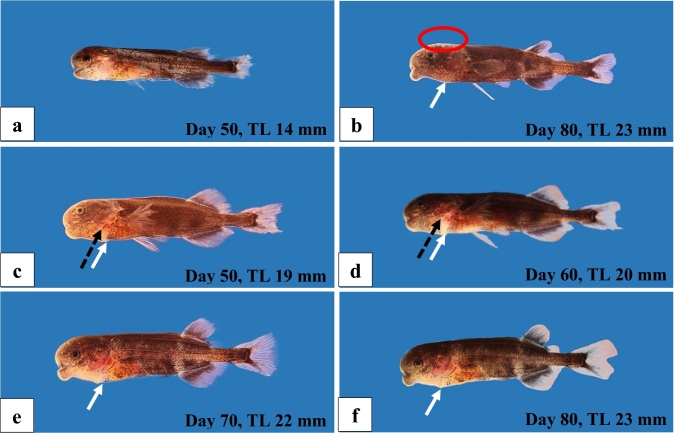
Larval and juvenile F1-hybrids (*Gnathonemus petersii* ♂ × *Campylomormyrus compressirostris* ♀) with morphological abnormalities, which later on apparently caused the death of these fish. **a** A very slim larva with injured unpaired fins. **b** A slim juvenile with fungal infection on the head (*red circle*) and a swollen ventral region of the head (white arrows). Larva (**c**) and juveniles (**d–f**) with malformations in head region: abnormal developed opercula (black arrows) (**c, d**) and a swollen ventral region of the head (white arrows) (**c–f**)

In Fig. 5, we depict some larvae and juveniles showing abnormalities, which were apparently cause of their death. Figure 5a shows a larva, which did not feed well and was very meager. Figure 5b depicts a juvenile with a fungus infection on the head and a swollen ventral region of the head, which is also obvious in one larva (Fig. 5c) and the juveniles shown in Fig. 5d–f. Furthermore, malformed opercula were observed (Fig. 5c, d).

**Fig. 6:**
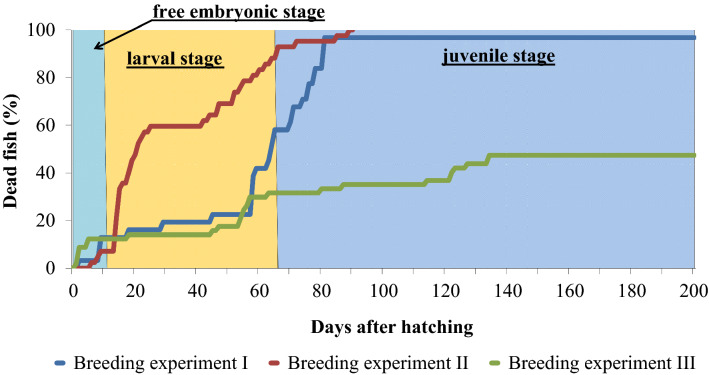
Death records of F1-hybrids (*Gnathonemus petersii* ♂ × *Campylomormyrus compressirostris* ♀) of three different breeding experiments recorded over an ontogenetic period of 200 days

The F1-hybrids died within different periods of the ontogenetic development and the survival rate of the F1-hybrids varied considerably in the three raising experiments, as did the periods during which these F1-hybrids died during ontogeny (see Fig. 6).

### Growth

**Fig. 7:**
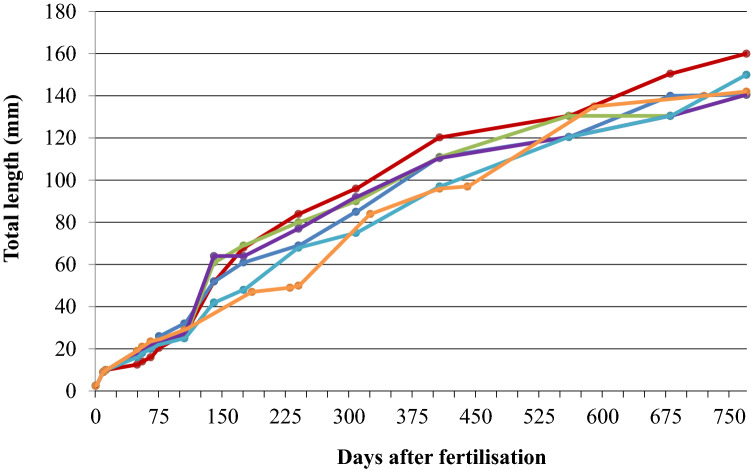
Growth curves of six individually raised F1-hybrids (*Gnathonemus petersii* ♂ × *Campylomormyrus compressirostris* ♀) over an ontogenetic period of 770 days

From six individually raised hybrids (one from the breeding experiment I and five from the experiment III), we obtained growth curves (Fig. 7). Up to day 100 at a TL of about 25 mm, there were no differences in the growth of the six individuals. Afterwards, growth rate increased and differences in the growth of the individuals were noticed. Between days 170 and 400, growth was nearly linear with a rate of about 7 mm per month. Thereafter, growth slowed down and on day 550 TLs of 120.5–130.5 mm were observed. On day 680 after fertilisation, TLs were 130.5–150.5 mm (average TL 136.5 mm). On day 770, the adult fish had achieved TLs of 140.5–160 mm (average TL 146 mm).

Seven fish, which were raised in the community tank, were measured two times during their ontogeny: on day 685 and 770, respectively. On day 685, these fish exhibited TLs of 95–130 mm (average TL 108 mm) and on day 770, TLs were 100–140 mm (average TL 118 mm). Thus, the fish of the community tank showed a slower growth compared to the individually kept fish.

Aggression and reduced food availability might have been the cause for this growth’s difference between fish raised individually vs. in the community tank.

### Sexual maturity and fertility

**Fig. 8:**
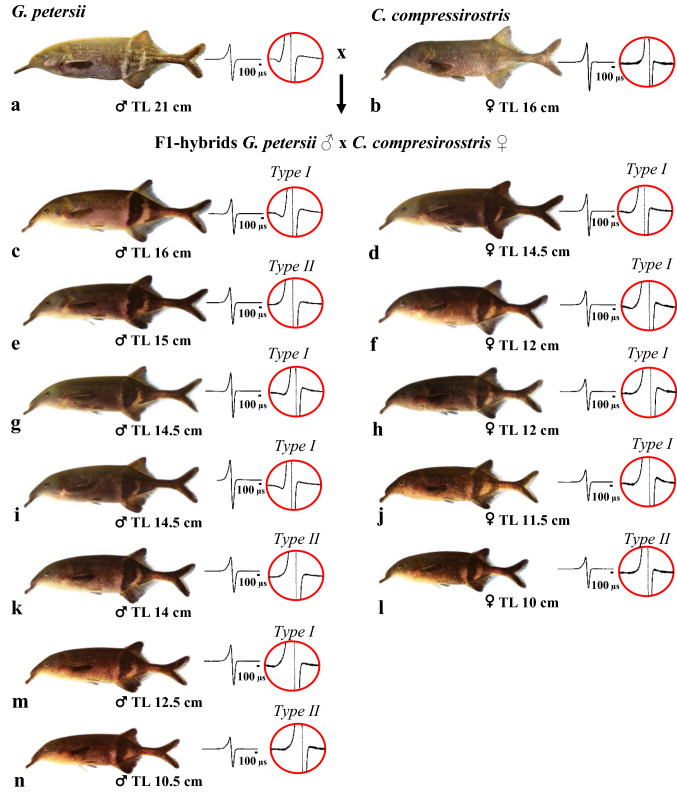
Morphology and EOD of parent species (*Gnathonemus petersii* ♂ and *Campylomormyrus compressirostris* ♀) and of their F1-hybrids. Males can be differentiated from the females by the modified anal fin (compare, e.g., **c** and **d**). The corresponding EODs are indicated at two different magnifications to depict the two types of EOD: type I (tetraphasic) and type II (biphasic resp. triphasic)

The morphology and the EODs of 12 fish, kept for months in a community tank, are shown in Fig. 8. Due to the sexual dimorphism at the anal fin (males have a larger and lobed anal fin) (compare Fig. 8c, d), we could identify seven males and five females. The smallest male had a TL of 10.5 cm and the smallest female measured 10 cm. Thus, the minimum size for sexual maturity for these intergenus F1-hybrids was about 10 cm. Apart from the differences at the level of the anal fin, F1-males and females looked quite similar and there were no differences in the colour pattern, both sexes of hybrids resembling the pigmentation of *G. petersii*.

14 additional F1-hybrids (eight males (16–18.5 cm of a TL) and six females (13.5–14.5 cm of a TL)) were kept together and a natural breeding experiment was conducted using the technique of imitation of the rainy season, which was successful for other mormyrids and their hybrids (Kirschbaum 1987, 2006; Kirschbaum and Schugardt 2002b, 2006; Nguyen et al. 2017; Korniienko et al. 2020). After some weeks, we noticed an increase in the volume of the body cavity in some females; however, spawning did not occur. We, therefore, conducted an AR experiment with four females and four males. From three females, we obtained in total 1144 eggs and some sperm liquid from three males. However, we did not obtain fertilised eggs from a batch of 366 eggs selected for AR.

### EOD ontogeny

**Fig. 9:**
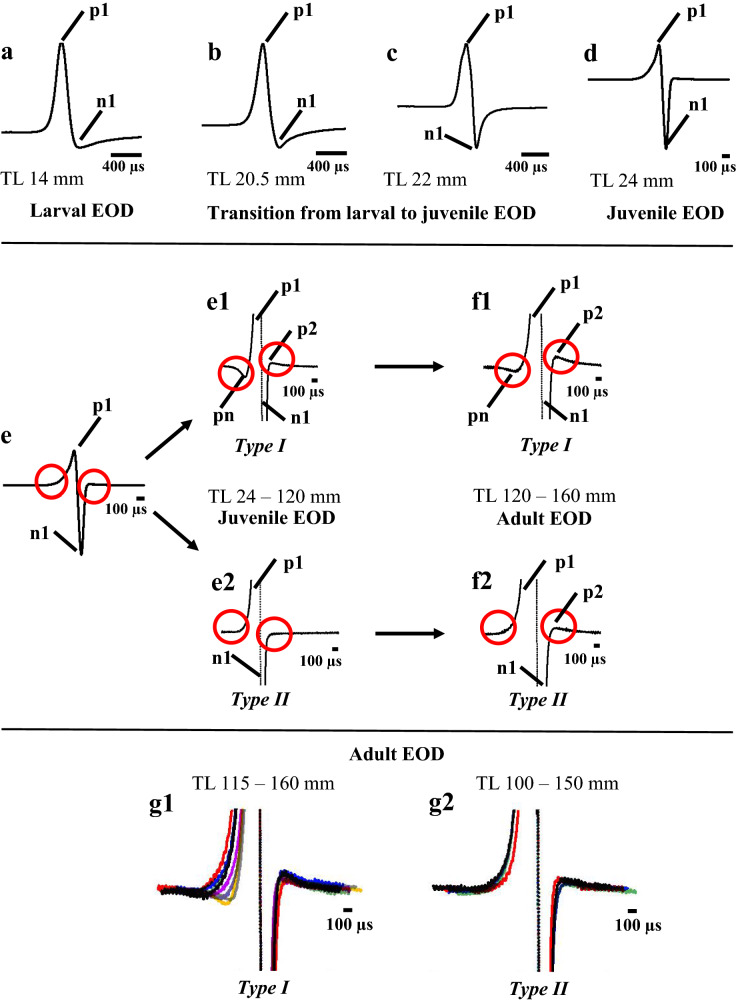
Ontogeny of the EOD of F1-hybrids (*Gnathonemus petersii* ♂ × *Campylomormyrus compressirostris* ♀). **a–d** Larval EOD with a large head positive phase (p1) and a smaller head negative phase (n1). **b, c** Transition from larval to juvenile EOD with an increasing amplitude of the head negative phase (n1). **d** Juvenile EOD. Note that larval and juvenile EODs have been recorded at different time bases (400 µs and 100 µs, respectively). **e, f** The two types of EOD (I, II), which occur at the juvenile stage (**e1, e2**) and remain unchanged up to adulthood (**f1, f2**). The different phases of the EOD are labelled in the high magnification: (p1) first head positive phase, (p2) small second head positive phase, (pn) small initial head negative phase and (n1) first head negative phase. Overlays of Type I EODs of eight specimens (**g1**) and Type II EODs (**g2**) of four specimens (see Fig. 8)

The EODs of the parent species and the EOD ontogeny of the F1-hybrids are shown in Fig. 8 and depicted in further detail in Fig. 9. The paternal species *G. petersii* (Fig. 8a) produces a short tetraphasic EOD (found at high magnification) with duration between 280 and 380 µs (Terleph and Moller 2003), whereas the maternal species *C. compressirostris* (Fig. 8b) shows a biphasic EOD of about 200 µs (Feulner et al. 2009a; Paul et al. 2015). The F1-hybrid larvae produce a biphasic EOD of about 3 ms duration with a large head positive phase and a smaller head negative phase (Fig. 9a). At around a TL of 20.5 mm, the amplitude of the head negative phase starts to increase (Fig. 9b) until the head negative phase is about twice as high as the head positive phase in 24 mm long fish (Fig. 9d). This is a typical juvenile EOD of *Campylomormyrus* species (Nguyen et al. 2017). The duration of this juvenile EOD (varying between 150 and 200 µs) has decreased compared to the larval EOD (compare Fig. 9a, d) with a duration of about 3 ms (duration measurement provided according to Westby and Kirschbaum 1977). At higher magnification, it becomes obvious that there are two EOD types: a tetraphasic type (Type I, Fig. 9e1) and a biphasic type (Type II, Fig. 9e2). Type I EOD persists up to adulthood (Fig. 9f1, g1); whereas the biphasic type may develop into a triphasic EOD, due to the appearance of a small second head positive phase (Fig. 9f2, g2). About 2/3 of the specimens showed a tetraphasic EOD type and 1/3 the triphasic EOD type (Fig. 8). The overlays of the two EOD types (Fig. 9g1, g2) show that there are only small differences in each of the two types. We did not find a sexual dimorphism in the EOD.

## Discussion

### Artificial reproduction (AR)

To obtain F1-hybrids between male *G. petersii* and female *C. compressirostris*, we performed three independent AR experiments (Table 1), for which we selected males and females from the breeding tanks. We chose four different males, which could be identified based on their TL and their anal fin features. The females could not be identified individually, but were selected based on their body volume: thick females indicated well-developed ovaries; this was confirmed through the release of ovulated eggs in all three reproduction experiments (Table 1). Despite the different origin of the gametes in the three experiments, the fertilisation rates were very similar (20.1–25.3%) and the number of hatched embryos did not vary much (86.1–97.7%). However, the survival rates of the F1-hybrids varied considerably: between 0% and 48.4% (Table 1). The possible cause for this result will be discussed in the paragraph below. For comparison, the fertilisation rate of the interspecific hybridisation between male of *G. petersii* and female of *C. rhynchophorus* was 35% and the hatching rate amounted to 68.6% (Kirschbaum et al. 2016). The fertilisation rates of the eight intraspecific *Campylomormyrus* hybrids showed a wide range varying between 19.7% and 94% (average 47.8%) and the hatching rates ranged between 16.4% and 99% (average 82.1%) (Kirschbaum et al. 2016). Thus, fertilisation and hatching rates of the two intergenus hybrids (*Gnathonemus* × *Campylomormyrus*) were not consistently different from the respective values of the intragenus hybrids (*Campylomormyrus*).

### Ontogenetic development and fertility

The F1-hybrids start to hatch on day 3 (about 70–72 h after fertilisation), exogenous feeding starts on day 11, the juvenile stage starts at a TL of ca. 21 mm (60–65 day-old-fish) and sexual maturity is achieved at a TL of 10–11 cm (Fig. 8). The embryos, the free embryos up to day 11 and the F1-hybrid larvae look like those of the parental species *C. compressirostris* (Nguyen et al. 2017) and *G. petersii* (Korniienko unpublished results). Furthermore, they are comparable to early ontogenetic stages of other mormyrid species (Kirschbaum 1987, 2006; Kirschbaum and Schugardt 2002a; Diedhiou et al. 2007; Nguyen et al. 2017), as species specific morphological features appear later in development. The transition from the larval to the juvenile stage occurs in the F1-hybrids at a TL of ca. 21 mm. This is similar to the development of *C. compressirostris*, *C. rhynchophorus* and *C. tshokwe* in which this transition occurs at a TL of about 20 mm (Nguyen et al. 2017). However, in smaller species this transition occurs at a smaller TL, e.g., in *Pollimyrus isidori*, which attains an adult TL of ca. 10 cm, it occurs at a TL of ca. 15–16 mm (Kirschbaum 1987). The minimum size for sexual maturity of the F1-hybrids was a TL of 10–11 cm (Fig. 8). *C. compressirostris* achieves maturity at a TL of 13–14 cm (Paul et al. 2015), in contrast to *G. petersii*, which attains sexual maturity at a TL of 15–16 cm (Korniienko unpublished data). Thus, the F1-hybrid *G. petersii* × *C. compressirostris* attains sexual maturity at a smaller size than the parental species. A similar phenomenon was observed in the F1-hybrid *C. tamandua* × *C. compressirostris* (Korniienko et al. 2020).

In our AR experiments with the F1-hybrids *G. petersii* × *C. compressirostris*, it was indeed shown that three females with a TL between 13.5 and 14.5 cm released a total of 1144 ovulated eggs, which proves that these females were fertile. From three males (sizes between 16 and 18.5 cm of a TL), used during this experiment, it was possible to obtain a liquid, which we interpreted as sperm; however, this liquid did not fertilise any egg in a batch of 366 eggs, which we selected from one of the three females. This was similar to the fertile F1-hybrids *C. tamandua* × *C. compressirostris*: not all the sexually mature males (identification based on the male-typical anal fin) chosen for the artificial breeding experiment gave sufficient sperm (Kirschbaum et al. 2016). The inter- and intragenus F1-hybrids, apparently, produce less sperm than the purebred species. To clarify this issue, histological investigations of the testis of the F1-hybrids would be helpful.

Despite a more transparent sperm liquid of the F1-hybrids, the fertile intragenus hybrids *C. tamandua* × *C. compressirostris* spawned naturally with an average fertilisation rate of 47.8% (Korniienko et al. 2020), which is quite high for mormyrid species (see e.g., Nguyen et al. 2017). As our first artificial breeding experiments with the intergenus hybrids *G. petersii* × *C. compressirostris* were not successful (see Results), it would be interesting to perform additional breeding experiments to find out, if they are also able to spawn naturally.

The fact that the intergenus F1-hybrids *G. petersii* × *C. compressirostris* are fertile indicates that the genetic difference between species of the two sister clades *Campylomormyrus* and *Gnathonemus* (Sullivan et al. 2000; Lavoué et al. 2003) are not large enough to prevent the occurrence of fertile F1-hybrids.

The morphology of the adult F1-hybrids *G. petersii* × *C. compressirostris* shows features of both parents, which is well seen in the morphology of the snout and the pigmentation of the trunk (Fig. 8). A more detailed analysis of this topic, based on landmarks and geometric morphometrics, is in progress (Amen unpublished results).

### Malformations and mortality

Malformations (Figs. 3–5) were observed during embryogenesis, after hatching in the free embryos, during the larval stage and at the beginning of the juvenile stage. These included abnormalities of the vertebral column, disturbances in the circulatory system, abnormalities of the heart region and of the ventral part of the body cavity. All these deficiencies finally led to the death of the specimens (Fig. 6) indicating deficiencies in the genetic inventory of these F1-fish.

Malformations were also described in intragenus *Campylomormyrus* hybrids (Baumgartner 2015; Elarbani 2017). However, in these hybrids typically only one of the abnormalities occurred at a time, whereas in the intergenus hybrids often two or even more abnormalities were observed in individual animals. For instance, some of the F1-hybrids of *C. tamandua* × *C. compressirostris* showed eye, snout or trunk abnormalities, yet they developed otherwise normally (Baumgartner 2015). Also in the intergenus hybrids *G. petersii* × *C. rhynchophorus*, malformations occurred more frequently and included two or more defects, such as a concave neck region in combination with missing eyes and disorder in the yolk (Elarbani 2017). The higher degree of malformations in the intergenus hybrids is likely related to the genetic differences between the two clades (see Lavoué et al. 2003).

In our study, the F1-hybrids died within different periods of the ontogenetic development (Fig. 6) apparently caused by genetic deficiencies. Deaths at the beginning of exogenous feeding might indicate problems with the digestive system concerning food uptake; deaths at the transition from the larval to the juvenile period occurred, when we switched to larger food items and moved the fish into larger tanks.

The time course of deaths in our three breeding experiments differed considerably (Fig. 6). We observed in both breeding experiments I and II symptoms, which we interpreted, based on our experience with raising mormyrid fish, as indication of diseases. This led to a complete death of all the hybrid-fish in experiment II and to the death of all fish (except one) in breeding experiment I. Still, we do not know why these diseases did not or only to a small part affect the fish of breeding experiment III. Probably, some uncontrolled aspects of rearing (i.e., food intake, injuries through aggression) are contributing to these differences.

#### EOD

The larval EOD of the F1-hybrids is biphasic with about 3 ms duration (Fig. 9a). Such a larval EOD is produced by both parental species (Kirschbaum et al. 2016; Nguyen et al. 2017) and is found in other *Campylomormyrus* (Nguyen et al. 2017) and several other mormyrid as well (Westby and Kirschbaum 1977, 1978; Baier et al. 2006; Werneyer and Kramer 2006). At a TL of 20.5 mm, the EOD of the hybrids starts to change and at a TL of 24 mm, the larval EOD is replaced by the juvenile EOD (Fig. 9b–d). This biphasic juvenile EOD has a different shape than the larval EOD and is shorter in duration (duration of about 150–200 µs). It is similar to the juvenile EOD of the parental species (Kirschbaum et al. 2016; Nguyen et al. 2017; Korniienko unpublished results). At higher magnification, it becomes apparent that there are two distinct juvenile EOD types: a tetraphasic EOD type (Type I, Fig. 9e1) and a biphasic EOD type (Type II, Fig. 9e2). Type II EOD further develops into a triphasic EOD (Fig. 9f2, g2). Applying structural–functional correlations provided by Bass (1986) for mormyrid EOs, this suggests anatomical differences in the EO of the F1-hybrids. Specifically, the first small initial head negative phase of Type I EOD and the subsequent large head positive phase would be compatible with penetrating small stalks, which originate at the caudal surface of the electrocyte and a rostral position of the main stalk. In contrast, type II EODs is indicative of a caudal position of the main stalk and caudally located, non-penetrating small stalks. These results suggest that the morphological design of the EO in the paternal *G. petersii* (rostral position of the main stalk and penetrating small stalks; Bruns 1971) has been transferred to the F1-hybrids. This was not the case in the hybrids of the cross *G. petersii* × *C. rhynchophorus* (Kirschbaum et al. 2016; Elarbani 2017). The split into two EOD types (Fig. 9e1–g1, e2–g2) in our sample indicates that this feature of the *G. petersii* EO is only inherited by ca. 65% of the hybrids (see Fig. 8). Heterozygous genetic background of the parental species, controlling the morphology of the EO, might be responsible for this divergence. Another explanation could be that, during early ontogeny, the structure of the hybrid EO is not yet fixed and underlies epigenetic influences in the one or the other direction.

Several recent studies in *Campylomormyrus* hybrids (Kirschbaum et al. 2016) and in wild populations of *Paramormyrus* (Gallant et al. 2011; Picq et al. 2020) argue for a further diversity in EO design in specimens with EODs containing an initial head negative (pre-potential) similar to Type I:

1. Hybrids of *C. tamandua* and *C. compressirostris* feature electrocytes with double penetrations but caudally located stalks (Kirschbaum et al. 2016). This is comparable to the EO in *Stomatorhinus corneti* (Bass 1986).
2. Hybrids of *C. tamandua* and *C. tshokwe* feature an EO of mixed morphology, where inside the column of electrocytes some cells possess a rostral and some a caudal position of the main stalk both featuring penetrations (Kirschbaum et al. 2016). Heterogeneously organised EOs were so far only reported in some populations of *Paramormyrops kingsleyae* from Gabon (Gallant et al. 2011; Picq et al. 2020).
3. In *Paramormyrops kingsleyae*, there is significant geographic variation in electric signal waveforms with some specimens exhibiting initial head negative (pre-potentials)–similar to Type I EODs in our hybrids–and others lacking them completely–similar to our Type II EODs. In *Paramormyrops*, the magnitude of the first head negative phase of the EOD (called “P0”) positively correlates with the number of penetrations per area of electrocytes (Gallant et al. 2011).

Further studies are needed to investigate the morphological basis of EOD diversity in the intergenus F1-hybrids. Picq et al. (2020) have demonstrated that *Paramormyrops kingsleyae* is capable of distinguishing between EODs with pre-potential and without. This suggests that this kinds of EOD variation could be a cue for assortative mating (Picq et al. 2020). Such a mixture of two EOD types is also found in our intergenus F1-hybrids. The breeding experiment to obtain natural spawnings with the F1-hybrids failed. Possibly, because the individuals with identical EODs could not segregate well enough in the breeding group in the restrictive space of the breeding conditions. Further behavioural experiments are necessary to assess this issue.

### Species stability in mormyrid fish

Hybrids are commonly observed in fishes and occur in more than 19.7% of the fish families worldwide (Nelson 1994). In marine species, hybrids are widely found in the families belonging to the Atherinomorpha and Percomorpha (Schwartz 1972, 2001). Hybrids in freshwater fish have been documented in 30 families (Schwartz 1981, 2001). Molecular genetic studies have repeatedly shown that hybridisation in fish is a common phenomenon and is often detected as an ancient introgression (Schliewen and Klee 2004; Herder et al. 2006; Schwarzer et al. 2012a, 2012b; Meier et al. 2017; MacGuigan and Near 2019). In the freshwater family of the Mormyridae with more than 200 species (Lavoué et al. 2003), natural hybrids are only rarely observed. Natural hybridisation has been found only in the genus *Paramormyrops*, which contains 22 species (Lavoué et al. 2008): hybrids were observed between morphs of the *Paramormyrops magnostipes* species complex characterised by differences of their electric organ discharge (Arnegard et al. 2005). In *Paramormyrops kingsleyae*, natural hybrids were observed among morphs occurring in geographic proximity (Gallant et al. 2011). Sullivan et al. (2004) discuss past introgression (i.e., hybridisation) in *Paramormyrops* inferred from mitochondrial data. Within *Campylomormyrus*, mitochondrial and single locus nuclear data do not reveal any sign of hybridisation/introgression (Feulner et al. 2007; Lamanna et al. 2016), while recent genome-wide Single-Nucleotide Polymorphism (SNP) data point towards the possibility of one or two ancient introgression events among species of this genus (Canitz, Kirschbaum and Tiedemann unpublished data).

The AR experiments in the mormyrid fish (Kirschbaum et al. 2016; Korniienko et al. 2020; this paper) have documented a high potential in mormyrid fish to generate fertile hybrids, such that postzygotic isolation seems weak or even absent. In contrast, natural hybrids seem to be rare (see above), pointing towards effective prezygotic isolation. Indeed, the EODs are very diverse and serve as reproduction isolation barriers, as association with conspecifics is strongly preferred (e.g., Feulner et al. 2009a; Gallant et al. 2011; Nagel et al. 2018; Picq et al. 2020). Such close contact is essential during spawning, as the male’s anal fin forms a pouch into which eggs and sperm are released (Crawford et al. 1986; Kirschbaum 1987). As the mormyrid sperm is lacking a flagellum (Mattei et al. 1972; Pecio 2020), free sperm are rarely available for fertilisation of eggs of other mormyrid species. EOD-based mate choice and the peculiarities of sperm and the fertilisation process apparently act in conjunction as very effective prezygotic isolation mechanisms leading to the high species stability of the mormyrid fish, despite of an apparent lack of postzygotic isolation.

## Conclusions and outlook

The F1-hybrids *G. petersii* × *C. compressirostris* are fertile and this fact shows that the genetic differences between the two sister clades *Campylomormyrus* and *Gnathonemus* (Sullivan et al. 2000; Lavoué et al. 2003) are not very large. Therefore, it would be interesting to perform additional AR experiments or to obtain natural spawning to obtain F2-fish and to follow their ontogenetic fate. Behavioural experiments with the F1-hybrids could indicate: (1) if the hybrids are able to distinguish between their own discharge and those of the parental fish; and (2) if this information is used for mate choice and could ultimately lead to reproductive isolation.

## Abbreviations

EO: Electric organ
EOD: Electric organ discharge
TL: Total length
AR: Artificial reproduction
